# Natural Mutations Affect Structure and Function of gC1q Domain of Otolin-1

**DOI:** 10.3390/ijms22169085

**Published:** 2021-08-23

**Authors:** Rafał Hołubowicz, Andrzej Ożyhar, Piotr Dobryszycki

**Affiliations:** Department of Biochemistry, Molecular Biology and Biotechnology, Faculty of Chemistry, Wrocław University of Science and Technology, Wybrzeże Wyspiańskiego 27, 50-370 Wrocław, Poland; andrzej.ozyhar@pwr.edu.pl

**Keywords:** analytical ultracentrifugation, C1q, calcium binding proteins, circular dichroism, genetic variation, otoconia, otolin-1, OTOL1, site-directed mutagenesis, thermal shift assay

## Abstract

Otolin-1 is a scaffold protein of otoliths and otoconia, calcium carbonate biominerals from the inner ear. It contains a gC1q domain responsible for trimerization and binding of Ca^2+^. Knowledge of a structure–function relationship of gC1q domain of otolin-1 is crucial for understanding the biology of balance sensing. Here, we show how natural variants alter the structure of gC1q otolin-1 and how Ca^2+^ are able to revert some effects of the mutations. We discovered that natural substitutions: R339S, R342W and R402P negatively affect the stability of apo-gC1q otolin-1, and that Q426R has a stabilizing effect. In the presence of Ca^2+^, R342W and Q426R were stabilized at higher Ca^2+^ concentrations than the wild-type form, and R402P was completely insensitive to Ca^2+^. The mutations affected the self-association of gC1q otolin-1 by inducing detrimental aggregation (R342W) or disabling the trimerization (R402P) of the protein. Our results indicate that the natural variants of gC1q otolin-1 may have a potential to cause pathological changes in otoconia and otoconial membrane, which could affect sensing of balance and increase the probability of occurrence of benign paroxysmal positional vertigo (BPPV).

## 1. Introduction

C1q superfamily encompasses short chain collagen-like proteins engaged in a wide variety of biological processes: immune recognition (C1q) [1], metabolic control (adiponectin) [2], endochondral ossification (collagen X) [3], formation of subendothelial and subcorneal matrices (collagen VIII) [4], cell adhesion in the retinal pigment epithelium (RPE) (Complement C1q tumor necrosis factor-related protein 5—C1QTNF5) [5] and more. Over the years, many disease causing mutations of the proteins from the C1q superfamily were detected. Many of them involve the globular C-terminal domain (gC1q), which is responsible for trimerization, which is usually Ca^2+^-dependent, and for interactions with the macromolecular ligands. Here, we focus on the missense mutations, which result in a substitution of a single amino acid. Clinically important mutations also involve frameshifts, which have more pronounced effects, insertion–deletion polymorphisms (indels), which result in excision or insertion of a DNA fragment, and mutations involving non-coding sequences, for example introns [6,7]. Typically, the pathogenic missense mutations of the gC1q domain interrupt trimerization, which results in the inability to form biologically active multimers and results in the lack of protein secretion or, in milder cases, secretion of defective, incorrectly folded protein. C1q protein, which initiates a classical complement pathway upon recognition of immune ligands, is a hexameric assembly of heterotrimers composed of chains A, B and C. G244R variant of chain B is associated with C1q deficiency, a rare genetic disease associated with systemic lupus erythematosus and increased susceptibility to bacterial infections [8]. In the case of adiponectin, R112C and I164T are examples of variants, which impair trimerization of adiponectin and secretion of the protein into circulation, which leads to reduced adiponectin levels and ultimately to a diabetic phenotype [9]. In the case of collagen X, various mutations in the gC1q domain (conventionally called NC1 for collagens) are associated with Schmid metaphyseal chondrodysplasia (spondylometaphyseal dysplasia), a rare genetic disease characterized by short stature, long bone growth abnormalities and waddling gait [10,11,12,13]. S163R variant of C1QTNF5 is involved in pathogenesis of late-onset retinal macular degeneration due to the weakening of the intracellular connections in RPE mediated by C1QTNF5. Moreover, mutated C1QTNF5 has decreased stability leading to its aggregation, which contributes to local tissue damage [5].

Otolin-1 is a protein from the C1q superfamily, which is a crucial component of the otoconial membrane and organic matrix of otoconia. Otoconia are small, numerous calcium carbonate biominerals, which appear as “ear dust” embedded in a gelatinous membrane. They are formed before birth. The otoconial membranes are connected to the hair cells of the sensory epithelia in the utricle and saccule, which are part of the vestibule in the inner ear. Aggregated otoconia move in response to the movements of the body, contributing together with semicircular canals to the sense of balance [14]. Interestingly, fish have analogous biominerals, otoliths, which in contrast are large, grow continuously during life and are involved in hearing [15,16]. Otolin-1 was first indirectly found through comparative analysis of amino acid content of organic matrices of otoliths from many species of fish, which showed exceptional conservation of amino acid composition and high content of hydroxyproline [17]. The *OTOL1* gene was cloned in 2002 for chum salmon *Oncorhynchus keta* [18] and in 2010 for mouse [19] and since then, sequences of otolin-1 from other organisms were inferred from homology.

Although the protein was cloned nearly 20 years ago, still only limited information is known regarding its structure and function in the inner ear. Ablation of otolin-1 in zebrafish resulted in formation of detached, often fused otoliths [20]. There are no reports showing the effects of knockdown of otolin-1 in mammals such as mice. Otolin-1 interacts with otoconin-90 (Oc90), another abundant otoconial matrix protein, through the globular gC1q domain and collagen-like domain [19,21]. Together with Oc90, it influenced the formation of calcite in vitro, which led to formation of barrel-like shape crystals resembling natural otoconia instead of rhombohedral, which appear in the absence of proteins with biomineralization activity. Otolin-1 and Oc90 had distinct effects on formation of calcite. Oc90 seems to increase the nucleation rate of calcium carbonate and inhibit growth of the crystals, whereas otolin-1 increased the rate of growth of the crystals. Nevertheless, such artificial otoconia were much larger than the natural biominerals, therefore the mechanisms of their synthesis in vivo depend on additional factors. In the same study, it was also shown that otolin-1 can form a hexagonal, fibrillary matrix, which predisposes it to form an organic scaffold of otoliths and otoconia [22]. It is important to note that in nature, not only calcite, the most stable polymorph of calcium carbonate, is produced, but aragonite, vaterite and amorphous calcium carbonate are found in the biominerals [23]. Otoliths of teleost fish are a good example, as they may contain aragonite or vaterite, depending on the species and growth conditions of the fish [16,24]. For rainbow trout (*Oncorhynchus mykiss*), it was shown that the high molecular weight aggregate extracted from the otolith matrix, which contained otolin-1, is necessary for formation of aragonite—a native polymorph of calcium carbonate. However, otolin-1 alone was not enough to drive the formation of aragonite [25]. Biomineralization of otoconia and otoliths is therefore a complex process, which depends on otolin-1, other proteins such as Oc90, and multiple other factors.

In our previous studies on the gC1q domain of otolin-1, we showed that it can form trimers; however, Ca^2+^ are required to form stable oligomers. We discovered that gC1q domain of human otolin-1 (hOtolC1q) forms stable oligomers at lower Ca^2+^ concentrations than the zebrafish analog, dOtolC1q, which relates to the differences in composition of endolymph in mammals and fish [26]. The mechanism of trimerization of hOtolC1q involves neutralization of repulsive charge at the axis of a trimer, which normally occurs due to binding of Ca^2+^ [27]. In this work, we analyzed the influence of identified natural variants of hOtolC1q on ability to form stable trimers and respond to increasing concentration of Ca^2+^, which is crucial for function of otolin-1 as an otoconial matrix protein and a constituent of the otoconial membrane. We were able to classify the variants according to the extent of their influence on the structure of hOtolC1q, and our results will enable to interpret clinical symptoms, which could be associated with the occurrence of mutations in *OTOL1* gene. We hypothesize that the mutations can disrupt the delicate homeostasis of otoconia and contribute to earlier occurrence of pathologies such as benign paroxysmal positional vertigo (BPPV).

## 2. Results and Discussion

**Figure 1:**
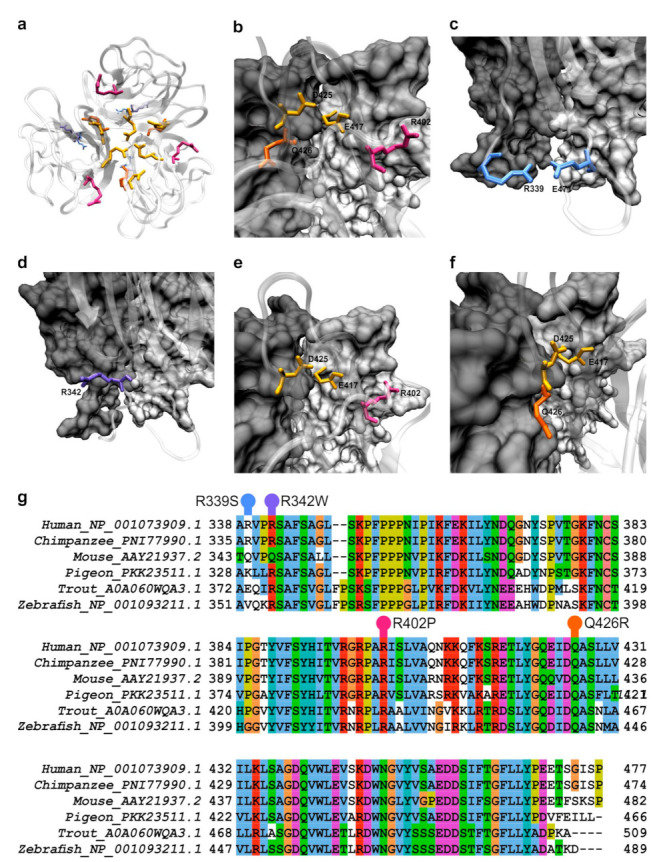
Structural and evolutionary context of the analyzed variants of hOtolC1q. (**a**–**f**) SAXS model of trimeric hOtolC1q with residues shown as sticks: R339S in light blue, R342 in deep blue, R402P in magenta and Q426R in orange. The E417 and D425 residues forming a Ca^2+^ binding site are shown in yellow. In **a**, all protomers are shown in a “cartoon” representation, in (**b**–**f**), one protomer is shown as translucent protein backbone, two others as light and dark gray protein surface projections. (**a**)—overall view of the gC1q trimer, (**b**)—Ca^2+^ binding site at the top of the trimer, (**c**)—R339, near the base of the trimer, with adjacent E471, (**d**)—R342 near the contact surface of the protomers, (**e**)—R402, and (**f**)—Q426, both near the Ca^2+^ binding site and the contact surface of the protomers. The visualizations were made using VMD [28]. (**g**)—Multiple sequence alignment showing the conservation of the gC1q domain of otolin-1 among the classes of the vertebrates. Investigated residues are highlighted above the alignment. The alignment was done using ClustalX [29] and visualized using Jalview [30].

During the database search, we found many single nucleotide variants (SNVs), including two single nucleotide polymorphisms (SNPs—SNVs with prevalence in the population of 1% or more [7]) in human *OTOL1* gene fragment encoding gC1q domain of otolin-1 (hOtolC1q). Then, we checked the position of affected residues in the small angle X-ray scattering (SAXS)-derived model of hOtolC1q trimer [26] and drew suppositions, how the mutations could affect structure and function of hOtolC1q. E470A (rs3921595) SNP was present in nearly 50% of sequencing reads. Since we suspected that due to its acidic properties E470 could contribute to a Ca^2+^ binding site, it was a subject of our previous analysis [27]. R339S (rs540167726) is a rarer SNP with maximal frequency of 2.5%. In the primary sequence, R339 is near the beginning of the gC1q domain and is placed at the base of a trimer (Figure 1a). It is modeled adjacent to E471 (Figure 1c), therefore it can form stabilizing ionic and hydrogen interactions; however, the importance of these interactions may be minor, as R339 is often replaced by other residues even in mammals (mouse as an example in Figure 1g, more examples in the Supplementary File S1). Although R339 is poorly conserved between the classes, we were interested how the substitution would affect hOtolC1q. Overall, this SNP was predicted to be neutral (Table 1). Out of the rarer variants of hOtolC1q, which were identified in multiple sequencing reads, R342W (rs200878802), R402P (rs760999493) and Q426R (rs1243409251) seemed to have a potentially significant impact. Side chain of R342 is exposed to the solvent near the boundary between the gC1q protomers (Figure 1d). Wider comparison of the mammalian sequences of otolin-1 showed that this residue is often substituted with glutamine (murine example in Figure 1g), even in apes (Supplementary File S1). However, substitution with tryptophan would have much more pronounced effect compared to glutamine, as it would introduce a hydrophobic aromatic moiety in place of a hydrogen bond donor/acceptor. This could affect the formation of trimers and modify the surface properties of the protein. R402 is located in the middle of a β-strand adjacent to a strand containing E417, which together with D425 forms a known Ca^2+^ binding site (Figure 1e). The side chain of R402 is predicted to be at the trimerization surface. Thus, substitution of this residue with proline could have a very strong detrimental effect on folding of the gC1q domain and binding of Ca^2+^. The malformation of the β-strand could propagate further, affecting the whole 10 β-barrel assembly typical for the C1q superfamily of proteins, especially near the Ca^2+^ binding site. Moreover, the substitution could affect the interactions between the protomers. Q426 follows D425 in the sequence, and its side chain is predicted to be at the trimerization interface (Figure 1f). Thus, substitution to arginine could affect the binding of Ca^2+^ and trimerization of the gC1q domain, although the effect should be weaker than for R402P.

The computational predictions accompanying the entries in the Ensembl database and independently conducted by us using SNPMuSiC suite (Table 1) suggested that all the rarer variants could be deleterious. The differences between the predictions obtained using different algorithms are too large to propose a relative degree of severity of the variants. CADD and MetaLR predictors gave results inconsistent with the other algorithms, as they did not differentiate the variants to benign or deleterious. However, as for REVEL and Mutation Assessor, CADD and MetaLR scores for R339S were lower than for the other variants, therefore it provides a rationale to differentiate this mutation as milder than the others. Predictions of varying severity of the mutations provided a motivation to produce the mutated hOtolC1q variants and subject them to analyses, which would reveal how the mutations affect the solution structure and Ca^2+^-dependent trimerization of hOtolC1q.

**Figure 2:**
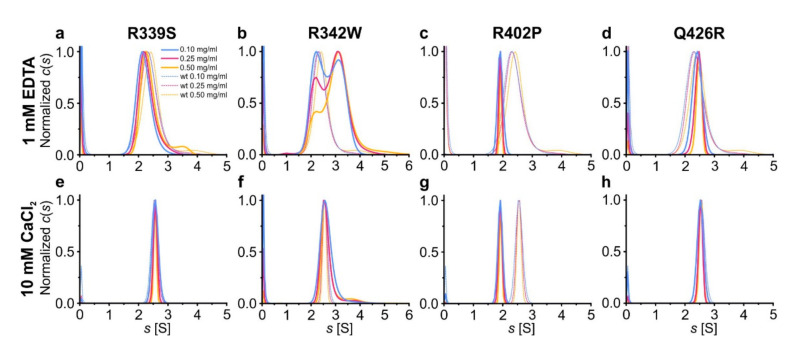
Influence of the mutations on oligomerization of hOtolC1q. Variants of hOtolC1q: (**a**,**e**) R339S, (**b**,**f**) R342W, (**c**,**g**) R402P, (**d**,**h**) Q426R were subjected to sedimentation velocity analytical ultracentrifugation at concentrations of 0.1–0.5 mg/mL in the presence of (**a**–**d**) 1 mM EDTA or (**e**–**h**) 10 mM Ca^2+^. The *c*(*s*) distributions are shown as solid lines. The dashed lines in the background show the *c*(*s*) distributions calculated for wild type hOtolC1q.

The typical feature of the proteins from the C1q superfamily is trimerization, usually Ca^2+^-dependent [1,3,32,33,34]. We used sedimentation velocity analytical ultracentrifugation (SV AUC) to see how the mutations could affect the assembly of gC1q trimers of hOtolC1q. We conducted the experiment for protein concentrations in the range of 0.1 to 0.5 mg/mL to properly characterize weak self-interactions already observed for wild type hOtolC1q [26]. There, sedimentation coefficient distributions (*c*(*s*)) calculated for varying concentrations of hOtolC1q centrifuged in the absence of Ca^2+^ were wide, with peaks between 2.0 and 2.5 S, and shifted continuously with increasing concentration from lower to higher sedimentation coefficients. The effect was even more pronounced for the zebrafish analogue, dOtolC1q. This phenomenon is characteristic for loosely bound complexes, which associate and dissociate rapidly during the SV AUC experiment [35]. The oligomerization of hOtolC1q seems to occur sequentially and follow a formula:

(1) $$A_{n}+A⇌\;A_{n+1}$$

where *A* is a protein monomer and the superscript indicates the stoichiometry of the oligomer. Fast kinetics of association and dissociation result in observation of intermediate species with sedimentation coefficients and apparent molecular weights of hOtolC1q between dimer and trimer, and even between monomer and dimer for dOtolC1q. A tendency of Ca^2+^-free hOtolC1q to form heavy aggregates was also noted. When 10 mM Ca^2+^ were added, a conformational change occurred which led to stabilization of the trimers at all tested protein concentrations. The trimers appeared in the *c*(*s*) distributions as a sharp peak at 2.55 S. Ca^2+^ ions also diminished the tendency of hOtolC1q to form heavy aggregates. Here, the experiment for hOtolC1q was replicated to serve as a control and the *c*(*s*) distributions are shown in the background of plots in Figure 2. In the case of R339S, the equilibrium of oligomerization in the absence of Ca^2+^ was slightly shifted to lighter forms compared to hOtolC1q (Figure 2a, Table S1), which we interpret as destabilization of the gC1q trimer. Conversely, Q426R variant apparently stabilized the gC1q trimer in the absence of Ca^2+^, as the equilibrium was shifted towards heavier forms (Figure 2d, Table S1). Moreover, hOtolC1q Q426R did not form heavy aggregates in the absence of Ca^2+^. The apparent beneficial effect of this variant is especially interesting if we consider that the most algorithms predicted it to be the most damaging (Table 1). Additionally, 10 mM CaCl_2_ diminished the effects of R339S and Q426R variants, as the *c*(*s*) distributions were identical as for hOtolC1q, showing the presence of homogenous trimers with no heavier aggregates (Figure 2e,h). R342W mutation was in contrast damaging, as it predisposed hOtolC1q to form heavy aggregates both in the absence and in the presence of Ca^2+^ (Figure 2b,f) in a proportion higher than for wild type hOtolC1q. In the absence of Ca^2+^, we observed discrete populations of dimers and tetramers, with increased proportion of tetramers at higher protein concentrations. Apparently, for this mutant, when Ca^2+^ is absent, the oligomerization mechanism switches from sequential association of monomers to association of dimers:

(2) $$2\;A\;⇌\;A_{2}⇌\;A_{4}\ldots \;A_{n}$$

Interestingly, the Ca^2+^ apparently rescued the correct oligomerization mechanism, as described by the Equation (1), because trimers were found for hOtolC1q R342W with 10 mM Ca^2+^. However, Ca^2+^ did not completely protect hOtolC1q R342W from aggregation, as the aggregate trace was still detected. R402P mutation had the most striking effect on the oligomerization of hOtolC1q—this variant was dimeric both in the absence and in the presence of Ca^2+^ (Figure 2c,g). Dimerization of hOtolC1q R342W and R402P did not involve the unique cysteine residue present in hOtolC1q (Figure 1g), as *c*(*s*) distributions calculated for samples centrifuged with 1 mM DTT were identical to those obtained in the absence of the reducing agent (Figure S1).

**Figure 3:**
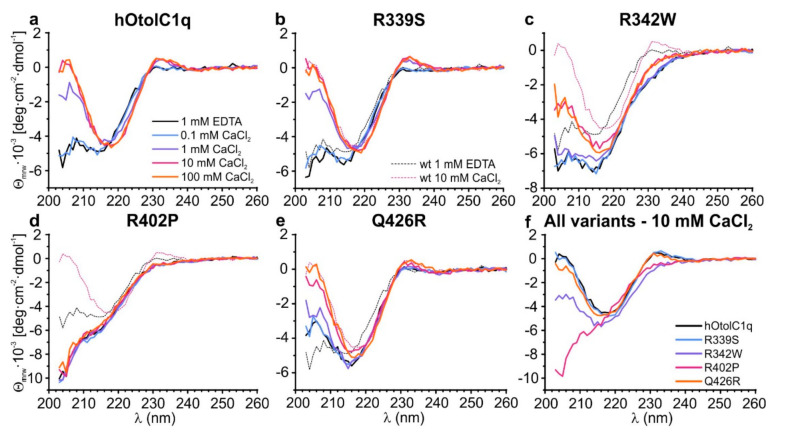
Changes in the secondary structure of (**a**) hOtolC1q introduced by the mutations: (**b**) R339S, (**c**) R342W, (**d**) R402P and (**e**) Q426R. Circular dichroism spectra were collected for 0.20 mg/mL proteins in the absence and in the presence of 0.1–100 mM Ca^2+^. The panel (**f**) contains a comparison of the spectra for all tested variants at 10 mM Ca^2+^.

SV AUC showed that the natural variants, especially R402P, had a major influence on the assembly of gC1q trimers of hOtolC1q. To gain more detailed insight into the structural change induced by the mutations, we applied circular dichroism spectroscopy (CD) (Figure 3) with the secondary structure estimation using CDPro (Figure S2). As for SV AUC, the experiment for hOtolC1q was replicated as a control (Figure 3a). The CD spectrum of hOtolC1q in the absence of Ca^2+^ indicates that the polypeptide chain is folded into β-sheets, as a negative band is present near 215 nm. The protein also contains a substantial amount of disordered regions, because the ellipticity decreases below 210 nm. There is also a notable signal attributed to aromatic side chains with a positive ellipticity maximum at 233 nm. In the presence of at least 1 mM Ca^2+^, structural change attributed to increase in β-strand content caused by binding of Ca^2+^ can be observed: position of the minimum shifts from 215 to 218 nm, and ellipticity is sharply increasing below 215 nm. Ellipticity near 233 nm also increased in response to added Ca^2+^ possibly due to structural rearrangements around the indole moieties of tryptophan side chains [26]. Similar features can be observed in the spectra of R339S mutant (Figure 3b). The band at 215 nm present in the absence of Ca^2+^ is slightly deeper than for the wild type hOtolC1q, but in the presence of 10 mM Ca^2+^, the spectra of R339S and the native form are identical (Figure 3b). This result is consistent with SV AUC, where small differences were also observed in the absence of Ca^2+^ and none in the presence of Ca^2+^. Similar changes appeared for the Q426R mutant (Figure 3e); however, the Ca^2+^-induced conformational change became apparent at 10 mM Ca^2+^ instead of 1 mM. This indicates that the substitution near the Ca^2+^-binding site weakened the affinity of hOtolC1q towards Ca^2+^. The spectra of hOtolC1q R342W do not have the positive band at 233 nm and have a deeper negative band at 215–218 nm (Figure 3c). Interestingly, 10 mM Ca^2+^ instead of 1 mM was required to induce the structural change here as well. This shows that the mutation at the base of a trimer, at the opposite side from the Ca^2+^ binding site, can have a pronounced effect on binding of Ca^2+^. As in the case of SV AUC, the most striking effect was noted for the R402P mutant, which was completely insensitive to the presence of Ca^2+^ (Figure 3d). The spectrum also shows no signal around 233 nm and a sharp decrease in ellipticity below 210 nm, which suggests that the degree of disorder compared to the wild type hOtolC1q was increased, probably due to the disruption of the β-strand containing R402, and possibly due to further alterations. Taken together, the CD spectra for hOtolC1q saturated with 10 mM Ca^2+^ (Figure 3f), which is biologically relevant since otolin-1 is present in the matrix of calcium carbonate otoconia, show that considering the secondary structure, R339S and Q426R mutations are benign, R342W is deleterious, and R402P may severely disrupt the function of hOtolC1q.

**Figure 4:**
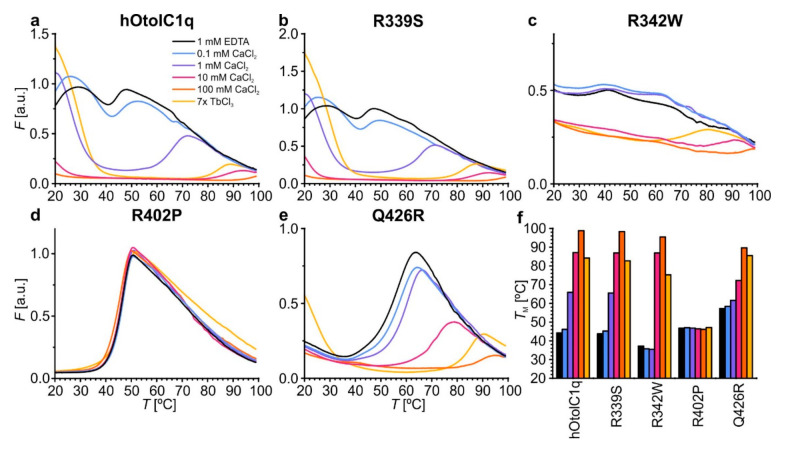
Changes in the thermal stability of hOtolC1q introduced by the mutations, analyzed by thermal shift assay (TSA). (**a**) Native hOtolC1q (control), (**b**) R339S, (**c**) R342W, (**d**) R402P and (**e**) Q426R. The *T*_m_ values are aggregated in the bar graph (**f**) and in Table S2.

An even more detailed view of the changes caused by the natural variants can be obtained using thermal shift assay (TSA). We previously used this technique to discover the striking stabilization of hOtolC1q with Ca^2+^, evidenced by transition temperature (*T*_m_) change from 40 to over 95 °C. The results were consistent with temperature-dependent changes in the CD spectra [26]. Using TSA, we also found striking effects of alanine mutations in the Ca^2+^ binding site of otolin-1, which did not always lead to the destabilization of the protein [27]. The experiment replicated here confirmed that native hOtolC1q was slightly stabilized with 0.1 mM Ca^2+^ and strongly stabilized at higher concentrations—the *T*_m_ increased to 66 °C in 0.1 mM Ca^2+^ and to more than 95 °C in 100 mM Ca^2+^ (Figure 4a). R339S was slightly, but consistently less stable than hOtolC1q—the *T*_m_ difference was near 2 °C under all tested conditions (Figure 4b). The slight decrease of *T*_m_ can be associated with ablation of interactions between R339 and E471 (Figure 1c). hOtolC1q was substantially destabilized by the R342W substitution, as the *T*_m_ was decreased to 37 °C in the absence of Ca^2+^ (Figure 4c). Moreover, this mutant was stabilized at 10 mM Ca^2+^, compared to 1 mM Ca^2+^ for wild-type hOtolC1q, which is consistent with the occurrence of the secondary structure change at 10 mM Ca^2+^. Ultimately, R342W had a stability similar to hOtolC1q at 10–100 mM concentrations of Ca^2+^, showing that Ca^2+^ mitigated the detrimental effect of the mutation. It was also noticeable that the fluorescent signal of SYPRO Orange bound to R342W was much weaker than for other variants, and the transitions were not clear. Modification of the surface properties of hOtolC1q by the mutation apparently interfered with binding of the SYPRO Orange probe. Q426R and, to a lower extent, R402P, were more stable than hOtolC1q in the absence of Ca^2+^ (*T*_m_ of 57.2 °C and 46.8 °C, respectively, Figure 4d,e). However, the Ca^2+^ stabilized Q426R more weakly than hOtolC1q (*T*_m_ 72.2 °C compared to 86.9 °C at 10 mM Ca^2+^), and did not stabilize R402P at all. Together with the results of SV AUC, this shows that Q426R mutation is stabilizing at low concentrations of Ca^2+^, but detrimental at higher concentrations, albeit not damaging enough to prevent trimerization of hOtolC1q. The results of TSA are also fully compatible with the results of CD, which showed that R342W and Q426R mutants are less sensitive to Ca^2+^ than the native hOtolC1q. The summary of the *T*_m_ for all the tested variants is provided in Figure 4f and Table S2.

TSA also provides interesting insight into the extent of exposition of hydrophobic regions on the surface of a protein, which was exhibited for gC1q domains of C1q and collagen X [1,3]. Affinity of hOtolC1q to hydrophobic compounds in the native state is evident as SYPRO Orange emits a strong fluorescence before the protein becomes unfolded. The further increase of fluorescence attributed to denaturation appears upon heating, when hydrophobic regions from the core of the protein become exposed and accessible for SYPRO Orange (Figure 4a). This is equivalent to binding of 8-anilino-1-naphthalenesulfonic acid (ANS), a fluorescent probe used specifically to probe the affinity of proteins to hydrophobic compounds [36,37]. ANS was in fact used in a prototypical TSA experiment [38] before SYPRO Orange was adopted due to its superior compatibility with existing qPCR devices [39]. In the case of hOtolC1q, the exposition of hydrophobic side chains decreases upon binding of Ca^2+^, as 10–100 mM Ca^2+^ decrease the baseline fluorescence at 20 °C (Figure 4a). While R339S mutant shows similar behavior to hOtolC1q, the increase of fluorescence of SYPRO Orange during denaturation of the R342W is poor, decreasing the robustness of the analysis for R342W mutant (Figure 4c). Interestingly, R402P and Q426R mutations decreased the baseline fluorescence (in the case of R402P—to a background level), which shows that structural alterations caused by these mutations resulted in inaccessibility of the hydrophobic surface groups of hOtolC1q in the native state (Figure 4d,e). This could hamper the interactions with other macromolecules in the otoconial matrix or otoconial membrane and impair proper formation and anchoring of otoconia during the embryonic development [40]. Overall, beside the primary evidence of change of thermal stability, TSA contributes to the observations that mutations and binding of Ca^2+^ cause pronounced structural changes in hOtolC1q affecting the whole globular trimer.

**Figure 5:**
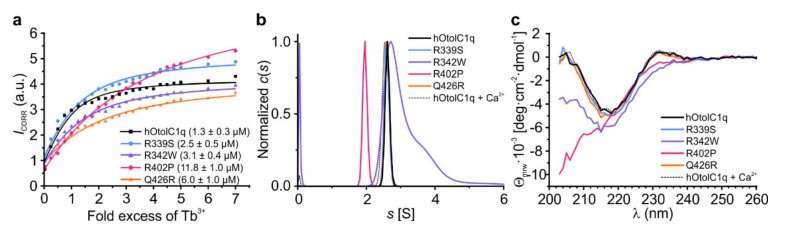
Binding of Tb^3+^ by hOtolC1q and its variants, analyzed by fluorometric titration, sedimentation velocity analytical ultracentrifugation and circular dichroism. (**a**) The results of a titration experiment, in which 3.7 µM proteins were treated with appropriate excess of TbCl_3_. The fluorescence intensity data were measured 15 min after addition of each portion of Tb^3+^, corrected for background fluorescence and fitted to a single binding site per monomer model. Apparent *K*_d_ values are given in the parentheses in the legend. (**b**) The *c*(*s*) distributions calculated from sedimentation velocity data obtained for 0.25 mg/mL (14.7 µM) hOtolC1q variants in the presence of 7-fold molar excess of TbCl_3_. The distributions for R339S and Q426R variants overlap with that calculated for hOtolC1q. Distribution of hOtolC1q with 10 mM Ca^2+^ is shown as a dashed line in the background for comparison. (**c**) The circular dichroism spectra recorded for 0.2 mg/mL (11.8 µM) hOtolC1q variants in the presence of 7-fold molar excess of TbCl_3_. Spectrum obtained for hOtolC1q with 10 mM Ca^2+^ is shown in the background as a dashed line.

The SV AUC, CD and TSA analyses showed that the mutations affected not only the solution structure of hOtolC1q, but also decreased (R342W, Q426R) or diminished (R402P) the ability of hOtolC1q to bind Ca^2+^. We sought to gain a more detailed insight into these effects by conducting a Tb^3+^ binding assay, which allows to estimate the relative affinity of the protein to Ca^2+^ [27]. In the case of hOtolC1q, the direct measurement of binding of Ca^2+^ was not possible due to the irreversible precipitation of the protein during decalcification procedure involving either incubation with EDTA/EGTA and exhaustive dialysis against a decalcified solution, or direct incubation with buffered metal-binding Chelex resin. As Tb^3+^ tend to strongly bind to the Ca^2+^-binding proteins [41,42], these ions could displace the trace Ca^2+^ from buffers and host cells and allow to conduct a comparative analysis of affinity of the proteins to Ca^2+^. We observed that all the mutations decreased the affinity of hOtolC1q to Ca^2+^: the dissociation constant (*K*_d_) was increasing in the order of hOtolC1q < R339S < R342W < Q426R < R402P (Figure 5a). The binding of Tb^3+^ was equivalent to Ca^2+^. hOtolC1q, R339S and Q426R responded to Tb^3+^ similarly as to Ca^2+^ by forming homogenous trimers (Figure 5b). Their CD spectra in the presence of Tb^3+^ were also identical as in the presence of Ca^2+^ (compare Figure 5c and Figure 3f). As expected, the structural changes occurred at low concentration of Tb^3+^, 35–82 µM, depending on the experiment. Interestingly, Q426R seemed to bind Tb^3+^ more preferentially than the other variants, as 35 µM Tb^3+^ stabilized it more strongly than 10 mM Ca^2+^. In the case of R342W, Tb^3+^ had an additional effect of intensifying the aggregation (compare Figure 5b with Figure 2f). Interestingly, despite the lack of responsiveness to Ca^2+^, R402P mutant seemed do bind Tb^3+^. Apparent affinity to Tb^3+^ was actually greater than for dOtolC1q, which responded to Ca^2+^ at higher concentrations than hOtolC1q [26,27]. Apparently, despite losing the ability to bind Ca^2+^, the R402P mutant retained some affinity to Tb^3+^. The binding seems to be non-specific though, as Tb^3+^ did not alter the secondary structure, induce trimerization or increase the thermal stability of the R402P mutant (Figure 4d and Figure 5b,c). Non-specific binding of lanthanide ions, including Tb^3+^, was identified for many proteins by X-ray crystallography [43]. Although the Tb^3+^ binding assay alone is not sufficient to determine the absolute affinity of a protein to Ca^2+^, it is useful for comparative analyses of the variants of the same protein from the same organism, when direct measurement of affinity to Ca^2+^ is not available. Here, CD and TSA were useful supplementary techniques. Moreover, SV AUC provided a mechanistic insight into the effects of mutations on the Ca^2+^-dependent assembly of hOtolC1q, and supported the observation that R402P mutant is effectively unable to bind Ca^2+^.

Although the availability of the phenotype data, which can be associated with specific genetic variants, is continuously increasing, the algorithms which depend solely on the protein sequences do not allow to reliably predict the effects of the mutations. From the variants of hOtolC1q clearly predicted to be deleterious: R342W, R402P and Q426R, Q426R seemed like a candidate for the most detrimental of the three. However, the R402P variant was clearly the most damaging for the protein. It is worth noting that this variant was correctly predicted as the most damaging by a model-dependent algorithm SNP MuSiC. This highlights the importance of structural studies of proteins involving not only atomic-level structure determination with nuclear magnetic resonance, X-ray crystallography or cryoelectron microscopy, but also molecular shape determination with less precise techniques such as SAXS or its sister method, small angle neutron scattering (SANS). Together with advanced computational 3D structure prediction methods, SAXS and SANS can give enough structural information to correctly predict the effects of the mutations on the structure and function of the proteins. This is especially important in the analysis of intrinsically disordered proteins, which lack a defined structure to a varying extent, and thus determination of their 3D structure at atomic resolution may be impossible [44]. In our case, CD and TSA gave detailed information regarding the effects of the mutations of the gC1q domain of otolin-1. SV AUC gave more general, but very important insight on a larger scale, as it showed how the trimer assembly and aggregation propensity of hOtolC1q were affected. This is an example of the advantage of SV AUC as a preferred method for analysis of mutated oligomeric proteins. Overall, the molecular arrangement of gC1q trimer seems to be resilient against relatively benign mutations, but severely affected by the extensive disruption of the secondary structure within the protomer (R402P) or by major modifications of solvent-exposed moieties (R342W).

R339S polymorphism of hOtolC1q is potentially benign, as it may affect structure and function of otolin-1 to a small extent and only at low concentrations of Ca^2+^. We noted similar effect for a prevalent polymorphism of otolin-1, E470A, which similarly to R339S slightly decreased the stability of hOtolC1q trimer in the absence of Ca^2+^ and slightly increased its tendency to form heavy aggregates [27]. Although these changes do not seem to be significant, they may negatively affect a decades long function of otolin-1 in the inner ear. According to the accumulated knowledge, otoconia and otoconial membrane do not regenerate, and the susceptibility of the otoconia to detach and incidentally accumulate in the semicircular canals leading to BPPV steadily increases during life [45,46,47]. Although it is normal that small amounts of otolin-1 leak from the labyrinth, patients suffering from BPPV have increased levels of otolin-1 in the serum [48,49]. It is important to note that beside the otoconial matrix, which is embedded in the solid calcium carbonate otoconium, otolin-1 is found in a fibrillary network interconnecting the otoconia [19,21,50], which makes it exposed to eventual pathological decreased level of Ca^2+^ in the endolymph. Destabilization of the otoconia and otoconial membrane, and resulting increased rate of release of otolin-1, may thus be a driving force of BPPV. Even the minor additional weakening of otolin-1 network caused by R339S and E470A mutations could accelerate the degradation of otolith organ enough to be a contributing factor to the earlier onset of BPPV, because they would make otolin-1 more sensitive to transient decreases in concentration of Ca^2+^ in the endolymph.

The rarer R342W and Q426R variants have strongly decreased the responsiveness of hOtolC1q to Ca^2+^ as they seemed to stabilize at approximately 10 mM of Ca^2+^ instead of 0.1–1 mM. Therefore, even in the healthy state with normal Ca^2+^ concentration in the endolymph (92–133 µM in guinea pig endolymph, possibly similar in humans) [51] these variants could weaken the network formed by otolin-1, induce early degradation of otolith organ and cause frequent BPPV at younger age. To remain stable, R342W and Q426R would require at least 1 mM Ca^2+^ in the endolymph, a concentration that is observed in hydropic ears serving as models for Ménière’s disease, which is characterized by the endolymphatic hydrops, attacks of vertigo and progressive hearing loss [51,52]. R342W and Q426R also modify surface properties of hOtolC1q, possibly interrupting protein–protein interactions in the otoconial matrix and otoconial membrane. R402P variant has a severe destabilizing effect on hOtolC1q, even preventing hOtolC1q from forming the trimers. As the network formed by otolin-1 seems to be interconnected by the globular heads of otolin-1 [22], such disruption would distort the protein matrix and cause a dysfunction of the otolith organ. However, lack of the clinical data related to the investigated variants of otolin-1 rule out the formulation of definitive conclusions. The results of our experiments should, therefore, bring attention to genetic variation of otolin-1 in patients with inner ear disorders, especially suffering from BPPV and other manifestations of imbalance in younger age. Protein–protein interactions in the otoconial membrane and in the otoconial matrix are another challenging area of research, which remains to be studied. Definite identification of the proteins involved, characterization of these interactions and effects of mutations would improve our understanding of the biomineralization mechanisms of otoconia and otoliths.

## 3. Materials and Methods

### 3.1. Accession Numbers

Human OTOL1 gene Ensembl accession ID: ENSG00000182447

Human otolin-1 Uniprot accession ID: A6NHN0

Human otolin-1 R339S SNP variant ID: rs540167726 (A > C)

Human otolin-1 R342W SNP variant ID: rs200878802 (C > T)

Human otolin-1 R402P SNP variant ID: rs760999493 (G > C)

Human otolin-1 Q426R SNP variant ID: rs1243409251 (A > G)

### 3.2. Key Resources

Synthetic cDNA encoding full-length human otolin-1 was codon optimized for *Escherichia coli* and provided by GeneArt (currently Thermo Fisher Scientific, Warsaw, Poland). Nucleotide primers were provided by Genomed (Warsaw, Poland). pQE-80L plasmid expression vector was from Qiagen (Hilden, Germany). *Escherichia coli* Top10 cells, *Dpn*I enzyme, DNA ladders, protein markers, and LB broth were from Thermo Fisher Scientific. One-fusion DNA Polymerase was from GeneOn (Ludwigshafen am Rhein, Germany; distributed by ABO, Gdańsk, Poland). BlueStain sensitive and SimplySafe stains were from EurX (Gdańsk, Poland). Agar, agarose, tris(hydroxymethyl)aminomethane (Tris), ethylenediaminetetraacetic acid (EDTA), carbenicillin, isopropyl β-D-1-thiogalactopyranoside (IPTG), NaCl, glycerol, 2-mercaptoethanol, imidazole, glycine, sodium dodecyl sulfate (SDS) and CaCl_2_ were from Carl Roth (Karlsruhe, Germany). *Escherichia coli* BL21(DE3) cells, TB broth, 4-(2-hydroxyethyl)-1-piperazineethanesulfonic acid (HEPES), phenylmethylsulfonyl fluoride (PMSF), DNase I, RNase A, terbium(III) chloride hexahydrate, xylenol orange disodium salt, dithiothreitol (DTT) and SYPRO Orange were from Sigma (currently Merck, Warsaw, Poland). Empty Tricorn and Superdex 200 Increase 10/300 GL columns were from (GE Healthcare Life Sciences, currently Cytiva, Warsaw, Poland). TALON^®^ Metal Affinity resin was from Takara Bio (Mountain View, CA, USA; distributed by Biokom, Janki, Poland).

### 3.3. Single Nucleotide Polymorphisms and Variants

Ensembl genome browser (https://www.ensembl.org/index.html, accessed 16 August 2021) was queried for known SNPs in human otolin-1 gene (OTOL1, ENSG00000182447). Boundaries of the gC1q domain were retrieved from Uniprot database (A6NHN0) as 338–477. The entries were accompanied by mutation severity predictions made using SIFT (https://sift.bii.a-star.edu.sg/, accessed 16 August 2021) [53], PolyPhen2 (http://genetics.bwh.harvard.edu/pph2/, accessed 16 August 2021) [54], CADD (https://cadd.gs.washington.edu/, accessed 16 August 2021) [55], REVEL (https://sites.google.com/site/revelgenomics/, accessed 16 August 2021) [56], MetaLR (https://sites.google.com/site/jpopgen/dbNSFP, accessed 16 August 2021) [57] and Mutation assessor (http://mutationassessor.org/r3/, accessed 16 August 2021) [58] tools. Additionally, for all investigated mutations, SNP MuSiC (https://soft.dezyme.com/, accessed 16 August 2021) tool was used to predict effects on protein stability [31]. Model of gC1q trimer, which was used as a template, was based on already published ensemble optimization method (EOM) analysis conducted on the basis of SAXS data [26]. Default parameters were used in all predictions. The structure model was visualized using VMD software (University of Illinois, https://www.ks.uiuc.edu/Research/vmd/, version 1.9.3, accessed 16 August 2021) [28].

### 3.4. Preparation of Mutated gC1q Genes

Synthetic cDNA of hOtolC1q, which was previously subcloned into pQE-80L plasmid expression vector [26], was used as a template in modified QuickChange^®^, which was conducted as described [59]. One-fusion DNA polymerase was used in the mutagenic polymerase chain reaction (PCR). For the calculation of the annealing temperatures of the primers, the concentration of KCl in the reaction mixture was assumed to be 0.1 M, as in the assay buffer of the polymerase. Plasmids were propagated in *Escherichia coli* TOP10 cells. Progress of the cloning was followed by agarose electrophoresis with SimplySafe stain. All mutated genes were analyzed by DNA sequencing (Genomed).

### 3.5. Protein Expression and Purification

*Escherichia coli* BL21(DE3) cells were chemically transformed by heat shock and grown on plates containing LB broth with 1.5% agar and 100 µg/mL carbenicillin at 37 °C overnight. Single colonies were picked and used to inoculate starter cultures containing 100 mL of TB broth with carbenicillin, which were incubated overnight at 37 °C, 200 rpm. Portions of 500 mL TB with carbenicillin were inoculated with 2% volume of starter culture and incubated at 29 °C, 200 rpm. After reaching the optical density at 600 nm of at least 0.5, cultures were cooled to 15 °C and the expression of the protein of interest was induced by 0.5 mM IPTG. The culture was continued overnight (16–18 h) at 15 °C, 200 rpm. Cells were collected by centrifugation at 5000× *g* at 4 °C for 15 min and resuspended in H10Na500G5 buffer (HEPES 10 mM, pH 7.0 (20 °C), NaCl 500 mM, glycerol 5% (*v*/*v*)) with freshly added 1 mM 2-mercaptoethanol. The cells were kept frozen at −80 °C.

Cell lysis was initiated by thawing in a room temperature water bath. After thawing, 0.2 mg/mL PMSF, 20 µg/mL DNase I and 20 µg/mL RNase A were added. The lysis was achieved by applying 10 sonication cycles for 30 s with 1 min breaks in a Cole-Parmer CPX 500 ultrasonic processor with a microtip and amplitude set at 35% (Cole-Parmer, Vernon Hills, IL, USA). The cell suspension was cooled in ice to maintain the temperature below 10 °C. Lysates were clarified by centrifugation at 18,500× *g* for 30 min at 4 °C and incubated with 1 mL TALON^®^ Metal Affinity resin for 1 h in a cold room (4–6 °C) in an orbital mixer set at 5 rpm. The resin was separated by centrifugation at 700× *g* for 5 min at 4 °C, washed with 20 bed volumes of H10Na500G5 (without the 2-mercaptoethanol), centrifuged again and packed in a glass Tricorn column. The column was connected to ÄKTA Avant chromatography system (GE Healthcare Life Sciences) with flow set at 1 mL/min. Contaminants were washed away with 20 bed volumes of H10Na500G5 and subsequently with 20 bed volumes of the buffer with 30 mM imidazole. Mutated hOtolC1q was eluted with the buffer containing 200 mM imidazole. The eluate was concentrated in Amicon Ultra centrifuge filters with 10 kDa cutoff (Merck) and subjected to gel filtration using Superdex 200 Increase 10/300 GL column operated at 0.75 mL/min with H10Na500G5 as a mobile phase. Pure fractions were identified by SDS-PAGE with acrylamide percentage of 4% in a stacking gel and 12% in a resolving gel in a Laemmli buffer system (Tris-glycine-SDS) (Figure S3) [60]. Pure protein samples were stored at –80 °C. For subsequent experiments, protein concentration was determined by measuring absorbance at 280 nm with elution buffer as a reference. The protein extinction coefficients and molecular weights were estimated using ProtParam tool (https://web.expasy.org/protparam/, accessed 16 August 2021) [61].

### 3.6. Tb^3+^ Binding Fluorescence

Binding of Tb^3+^ ions to hOtolC1q and its mutants was assessed using steady-state fluorescence. Terbium (III) chloride was dissolved in MilliQ water to a final concentration of approximately 0.5 M. Exact concentration of TbCl_3_ was determined by titration of diluted stock solution with EDTA in the presence of xylenol orange. Aliquots of diluted TbCl_3_ were added to 2 mL 3.7 µM protein solution in a 10 × 10 mm quartz SUPRASIL^®^ cuvette (Hellma Analytics, Müllheim, Germany) and incubated for 15 min at room temperature. Subsequently, fluorescence emission at 520-580 nm was recorded using an excitation wavelength of 280 nm using a Fluorolog-SPEX fluorimeter (HORIBA Scientific, Jobin-Yvon, Kyoto, Japan) equipped with a Peltier heating accessory set at 20 °C. The bandwidth was set at 5 nm for both excitation and emission monochromators. A cut-off filter absorbing below 350 nm was installed in the emission path. Obtained fluorescence intensities were processed and fitted to a model based on work by Gonzalez et al. [62,63]. Data analysis was conducted as described [27].

### 3.7. Circular Dichroism

Circular dichroism of 0.2 mg/mL proteins in H10Na500G5 with 1 mM EDTA, 0.1 mM CaCl_2_, 1 mM CaCl_2_, 10 mM CaCl_2_, 100 mM CaCl_2_ or 7-fold excess of TbCl_3_ was measured in 1 mm quartz SUPRASIL^®^ cuvettes (Hellma Analytics, Müllheim, Germany) using Jasco J-815 spectropolarimeter (Jasco, Easton, MD, USA) with a Peltier temperature control accessory set at 20 °C. The proteins were incubated with the additives at room temperature for at least 1 h before the measurements. The spectra were collected between 200 and 260 nm every 1 nm at scanning speed of 50 nm/min with five accumulations. Data, for which photomultiplier voltage was below 600 V, were analyzed. CD spectra of the proteins were corrected for buffer background signal and normalized for protein composition and concentration using an equation [64]:

(3) $$\theta_{mrw}=\frac{\theta \cdot MRW}{10\cdot c\cdot l}\;\left[\frac{\deg\cdot {\mathrm{cm}}^{2}}{\mathrm{dmol}}\right]$$

where $\theta_{mrw}$ is a mean residue ellipticity, θ—ellipticity [degrees], *MRW—*mean residual weight of a protein [g/mol], *c—*protein concentration [g/L] and *l—*optical pathlength of a cuvette [cm]. The secondary structure content was estimated using CDPro [65].

### 3.8. Analytical Ultracentrifugtion

Sedimentation velocity analytical ultracentrifugation (SV AUC) was conducted in a Beckman Coulter ProteomeLab XLI analytical ultracentrifuge (Beckman Coulter, Brea, CA, USA) with an An60Ti rotor and assembled cells with two-channel 12 mm charcoal filled Epon^®^ centerpieces and quartz windows, or sapphire windows for samples containing DTT. The proteins were analyzed at concentrations of 0.1, 0.25 and 0.5 mg/mL in H10Na500G5 with 1 mM EDTA or 10 mM CaCl_2_. Additional measurements were made for 0.25 mg/mL protein with EDTA and CaCl_2_ supplemented with 1 mM DTT. Effect of Tb^3+^ was analyzed by centrifuging 0.25 mg/mL protein with 7-fold molar excess of TbCl_3_. Assembled cells with the samples were preincubated in the ultracentrifuge for 3 h at 20 °C and then centrifuged at 50,000 rpm (approximately 200,000× *g* at the bottom of the cell) overnight. The absorbance scans at 280 nm were collected continuously with 0.003 cm resolution. The scans were time-corrected [66] and analyzed in SEDFIT (version, 16.1c, October 2018, available at https://sedfitsedphat.nibib.nih.gov/, accessed 16 August 2021) using a continuous *c*(*s*) distribution model [67] with at least 20 points per 1 S. Partial specific volumes of the proteins, densities and dynamic viscosities of the solvents were calculated using SEDNTERP (version 3.0.3, 14 March 2021, available at http://www.jphilo.mailway.com/download.htm, accessed 16 August 2021). Maximum entropy regularization with *p* = 0.95 was used. Simplex and Marquardt–Levenberg algorithms were alternately used until the RMSD converged. Among the results of the calculations were sedimentation coefficients (*s*), sedimentation coefficients corrected for water at 20 °C (s_20,w_), weight-averaged sedimentation coefficients ($\bar{s_{20,w}}$), apparent molecular weights (*MW*_app_) and frictional ratios (*f*/*f*_0_). *c*(*s*) distributions were visualized using GUSSI (version 1.4.2, 24 July 2018, available at https://www.utsouthwestern.edu/labs/mbr/software/, accessed 16 August 2021) [68] and Origin Pro 9.0 software.

### 3.9. Thermal Shift Assay

Thermal shift assay (TSA) was conducted as described [27]. Five µM solutions of the proteins in H10Na500G5 were supplemented with SYPRO Orange at concentration of 5× (hOtolC1q, R339S, R402P, Q426R) or 10× (R342W). The measurements were done in the presence of 1 mM EDTA, 0.1 mM CaCl_2_, 1 mM CaCl_2_, 10 mM CaCl_2_, 100 mM CaCl_2_, and 7-fold molar excess of TbCl_3_. Final sample volume was 20 µL. The samples and the non-protein controls (Figure S4) were aliquoted into a 96-well plate in triplicate, covered with optically clear foil and incubated at room temperature for at least 1 h before the measurements. Fluorescence of SYPRO Orange was measured using Applied Biosystems ImageQuant5 qPCR thermal cycler (Thermo Fisher Scientific) with optical filters set as x1-m3 (excitation at 470 ± 15 nm, emission at 587 ± 10 nm) between 20 and 99 °C during heating at 0.033 °C/s. The data were analyzed using Protein Thermal Shift software (Thermo Fisher Scientific). Transition temperatures (*T*_m_) were determined from the derivative of fluorescence with increasing temperature (d*F*/d*T*).

## Figures and Tables

**Table 1:** Selected known single nucleotide variants of hOtolC1q, their prevalence and predictions of deleteriousness. The data were retrieved from the Ensembl database, and for SNPMuSiC independently calculated based on the SAXS-derived model of hOtolC1q trimer (https://soft.dezyme.com/, accessed 16 August 2021) [31].

Variant dbSNP ID	Highest Population MAF	SIFT	PolyPhen	CADD	REVEL	MetaLR	Mutation Assessor	SNP MuSiC
R339S	0.025	0.5 (Tolerated)	0.097(Benign)	3 (Likely Benign)	0.129 (Likely Benign)	0.532 (Damaging)	0.268 (Low Impact)	−0.53 (Neutral)
R342W	1.159 × 10^−4^	0 (Deleterious)	0.880 (Possibly Damaging)	22 (Likely Benign)	0.326 (Likely Benign)	0.762 (Damaging)	0.904 (Medium Impact)	0.17 (Deleterious)
R402P	1.394 × 10^−4^	0 (Deleterious)	0.797 (Possibly Damaging)	22 (Likely Benign)	0.518 (Likely Disease Causing)	0.674 (Damaging)	0.792 (Medium Impact)	0.42 (Deleterious)
Q426R	4.643 × 10^−4^	0.02 (Deleterious)	0.969 (Probably Damaging)	23 (Likely Benign)	0.587 (Likely Disease Causing)	0.777 (Damaging)	0.758 (Medium Impact)	0.16 (Deleterious)

MAF—mean allele frequency, prevalence of the variant in the population. SIFT score has a scale from 0 to 1. Variants with scores below 0.05 are predicted to be deleterious. PolyPhen score has a scale from 0 to 1. Variants with scores up to 0.446 are predicted to be benign, from 0.447 to 0.908 to be possibly damaging, and with scores higher than 0.908 to be probably damaging. CADD provides a ranking with higher scores more likely to be deleterious, the customary boundary is set at 30. REVEL score ranges from 0 to 1 and variants with higher scores are predicted to be more likely to be pathogenic. MetaLR classifies the variants as ‘tolerated’ or ‘damaging’; a score between 0 and 1 is also provided and variants with higher scores are more likely to be deleterious. Mutation assessor gives a prediction, which is one of ‘neutral’, ‘low’, ‘medium’ and ‘high’, and the rank score, which is between 0 and 1 where variants with higher scores are more likely to be deleterious. For SNP MuSiC, positive score predicts the variant to be deleterious, negative to be neutral. SNP MuSiC also predicts solvent accessibility and effect on thermodynamic and thermal stabilities (results not shown).

## Data Availability

The data and materials underlying this article will be shared on request to one of the corresponding authors.

## Supplementary Materials

The following are available online at www.mdpi.com/article/10.3390/ijms22169085/s1, Supplementary File S1.pdf—multiple sequence alignment of mammalian sequences of gC1q domain of otolin-1 found during NCBI BLAST search. Supplementary File S2.pdf contains Figures S1–S4, Tables S1 and S2. Figure S1. Dithiothreitol (DTT) does not affect the oligomerization of hOtolC1q R342W and R402P. Figure S2. Estimation of the secondary structure content of hOtolC1q and its mutants. Figure S3. Purification of hOtolC1q and its mutants. Figure S4. Background fluorescence in the thermal shift assay. Table S1. Parameters derived from the sedimentation velocity analytical ultracentrifugation. Table S2. Transition temperature (*T*_m_) values (in °C) determined using the thermal shift assay.

## Abbreviations

ANS	8-anilino-1-naphthalenesulfonic acid
BPPV	benign paroxysmal positional vertigo
C1QTNF5	complement C1q tumor necrosis factor-related protein 5
CD	circular dichroism spectroscopy
*f*/*f*_0_	frictional ratio
dOtolC1q	gC1q domain of zebrafish otolin-1
DTT	dithiothreitol
EDTA	ethylenediaminetetraacetic acid
EOM	ensemble optimization method
gC1q	globular C-terminal domain
HEPES	4-(2-hydroxyethyl)-1-piperazineethanesulfonic acid
hOtolC1q	gC1q domain of human otolin-1
IPTG	isopropyl β-D-1-thiogalactopyranoside
*K*_d_	dissociation constant
*MW*_app_	apparent molecular weight
Oc90	otoconin-90
PCR	polymerase chain reaction
PMSF	phenylmethylsulfonyl fluoride
RPE	retinal pigment epithelium
s_20,w_	sedimentation coefficient corrected for water at 20°C
SANS	small angle neutron scattering
SAXS	small angle X-ray scattering
SDS	sodium dodecyl sulfate
SNP	single nucleotide polymorphism
SV AUC	sedimentation velocity analytical ultracentrifugation
*T*_m_	transition temperature
Tris	tris(hydroxymethyl)aminomethane
TSA	thermal shift assay
