# Supplementary material for: Natural Mutations Affect Structure and Function of gC1q Domain of Otolin-1
Source: Int J Mol Sci. 2021 Aug 23;22(16):9085. doi: 10.3390/ijms22169085 (PMC8396674; doi:10.3390/ijms22169085)
Supplement: Supplementary file 1 [file ijms-22-09085-s001.zip › Supplementary File 1.pdf]

NCBI Multiple Sequence Alignment Viewer, Version 1.20.0

Sequence ID	Alignment	Organism
	338 350 360 370 380 390 400 410 420 430 440 450 460 470 477	
Query_51791 (+)	ARVPRSAFSAGLSKPFPPPNIPIKFEKILYNDQGNYSPVTGKFNCSIPGTYVFSYHITVRGRPARISLVAQNKKQFKSRETLYGQEIDQASLLVILKLSAGDQVWLEVSKDWNGVYYVSAEDDSIFTGFLLYPEETSGISP	
NP_001073909.1 (+)		Homo sapiens
XP_004038004.2 (+)	V V	Gorilla gorilla gorilla
XP_526369.3 (+)		Pan troglodytes
XP_003830662.1 (+)		Pan paniscus
XP_002814299.2 (+)	A V	Pongo abelii
XP_032028518.1 (+)	A V Q G	Hylobates moloch
XP_003256290.3 (+)	A V Q	Nomascus leucogenys
XP_012302823.1 (+)	Q A D V S V I K	Aotus nancymaae
XP_002802984.2 (+)	A L V V L H I	Macaca mulatta
XP_011750982.1 (+)	A L V V L H I	Macaca nemestrina
XP_010334006.1 (+)	T Q A D V S V I K	Saimiri boliviensis bolivi...
XP_008006706.2 (+)	A V V L H I	Chlorocebus sabaeus
XP_003894926.3 (+)	A L V V L H I	Papio anubis
XP_033055485.1 (+)	A V A V E H I N T	Trachypithecus francoisi
XP_035134744.1 (+)	Q A L D V S V Q I K	Callithrix jacchus
XP_032117511.1 (+)	Q A D V S V R I K	Sapajus apella
XP_017392781.2 (+)	Q A D V S V R VI K	Cebus imitator
XP_010365030.1 (+)	A V A V E H I N T	Rhinopithecus roxellana
XP_032262168.1 (+)	Q A D V I A V H W R R H F I N P N	Phoca vitulina
XP_034845590.1 (+)	Q A D V I A V H W R R H F I N P N	Mirounga leonina
XP_024416125.1 (+)	A T V D VF A V S W V H I A R	Desmodus rotundus
XP_012638201.1 (+)	S Q A T D V T V R V I P P	Microcebus murinus
XP_029794513.1 (+)	V T D V D V A V W H I P S	Suricata suricatta
XP_036996853.1 (+)	AS V D VF A V S W V H IV P R	Artibeus jamaicensis
XP_004384939.1 (+)	G T A D VF S V A V P RP	Trichechus manatus lati...
XP_004835224.1 (+)	K Q VA D H D V M K T L LE	Heterocephalus glaber
XP_039097912.1 (+)	V T D V ED V A V H I P N	Hyaena hyaena
XP_032989907.1 (+)	Q G A A D VF A V A VQ W H I NV P	Rhinolophus ferrumequin...
XP_021558325.1 (+)	Q A D V I A V H W R R H F I N P N	Neomonachus schauins...
XP_028361075.1 (+)	A V D VF A V S W V H IV P R	Phyllostomus discolor
XP_026236157.1 (+)	KL Q A D D N A V K S D I L S PR	Urocitellus parryii
XP_027809096.1 (+)	KLLQ A D D N A V K D I L S K P	Marmota flaviventris
XP_008140474.1 (+)	T A V D VF A V S W H I R P	Eptesicus fuscus
XP_005338355.1 (+)	KL Q A D D N A V K D I LP I S P	Ictidomys tridecemlineatus
XP_025872233.1 (+)	V A A D V P A V WK R S H I L P D D	Vulpes vulpes
XP_041587459.1 (+)	V A A D V P A V WK K S H I L P D D	Vulpes lagopus
XP_006160023.1 (+)	S TSQ A T R N V S V H W I H L A P RT P	Tupaia chinensis
XP_032217618.1 (+)	AM A D V I A V W R H I P N	Mustela erminea
XP_027437825.2 (+)	QQ A D D V N I A V HV WDR R SAH F P N	Zalophus californianus
XP_027958902.1 (+)	Q A D D V N I A V HV WDR R SAH F P N	Eumetopias jubatus
XP_003358706.1 (+)	T T D V V A V Q W R H T I R NP	Sus scrofa
XP_037683161.1 (+)	GA A GV D V H F A V R H I G P M	Choloepus didactylus
XP_036902605.1 (+)	A A V D VF V A V S W V H I L P R	Sturnira hondurensis
XP_010602821.1 (+)	K Q VA D Y ED F A G I T H L L V PLEA	Fukomys damarensis
XP_030184823.1 (+)	L T D V V A V H I P N	Lynx canadensis
XP_004319915.2 (+)	Q A A D V A V Q W R H I K NP V	Tursiops truncatus
XP_033268146.1 (+)	Q A A D V A V Q W R H I K NP	Orcinus orca
XP_030701007.1 (+)	Q A A D V A V Q W R H I K NP	Globicephala melas
XP_004639916.1 (+)	VE Q VA T D V D A V M N H V L R P V K E	Octodon degus
XP_022269922.2 (+)	V A A D V P I A V WK R S H I L P D	Canis lupus familiaris
XP_023094334.1 (+)	L T D V V A V R H I P N	Felis catus
XP_032486839.1 (+)	Q A A D V A V Q W R H I K NP	Phocoena sinus
XP_003422243.1 (+)	G T A N D A H Q W L P PP	Loxodonta africana
XP_036302639.1 (+)	S A V D VF A V S W H T R MP	Pipistrellus kuhlii
XP_038301357.1 (+)	V A A D V P I A V WK R S H I L P D	Canis lupus familiaris
XP_040335474.1 (+)	L T D V V A V V R H I P N	Puma yagouaroundi
XP_025769832.1 (+)	L T D V V A V R H I P N	Puma concolor
XP_026968343.1 (+)	Q A A D V A V Q W R M H I K NP	Lagenorhynchus obliqui...
XP_036707624.1 (+)	A A D V A V Q SW R H I K NP	Balaenoptera musculus
XP_023601090.1 (+)	T A V R D VF A V S W H I R N P	Myotis lucifugus
XP_036208516.1 (+)	T A V D VF A V S W H I R N MP	Myotis myotis
XP_026916653.1 (+)	L T D V V A V H I P N	Acinonyx jubatus
XP_007187158.1 (+)	A A D VH A V Q SW R H I K NP	Balaenoptera acutorostr...
XP_011378203.1 (+)	Q VA V D VF R A V A R V H I N A P	Pteropus vampyrus
NP_001179533.3 (+)	A A D V A W R H T R D NPS	Bos taurus
XP_022431204.1 (+)	Q A A D V A V Q W R H I K V NP	Delphinapterus leucas
XP_006063019.1 (+)	Q A A D V A W R H T I R D NPS	Bubalus bubalis
XP_036075764.1 (+)	Q VA V D VF R A V A R V H I N A PE L	Rousettus aegyptiacus
XP_032691943.1 (+)	AM A D V I A V W H I P N	Lontra canadensis
XP_022358470.1 (+)	GAM A D V I A V W R H I P N	Enhydra lutris kenyoni
XP_039713458.1 (+)	Q VA V D VF R A V A R V H I NT A T P L	Pteropus giganteus
XP_001493990.1 (+)	P AA A A A DV DRV DA A V A A V S R VR H T V PET	Equus caballus
XP_036098209.1 (+)	P S A V D VF A V S W H R V P M	Molossus molossus
XP_040104039.1 (+)	A A A D V A W R L S H T I R D NP	Oryx dammah
XP_024619394.1 (+)	Q A A D V A V Q W R H I K NP	Neophocaena asiaeorie...
XP_020771029.1 (+)	A A D V V V W R H T I R D NL	Odocoileus virginianus t...
XP_031306095.1 (+)	T A R D V E A V W T R H I R NP	Camelus dromedarius
XP_028346655.1 (+)	A A D L A V Q W R H I K NP	Physeter catodon
XP_003794688.1 (+)	C Q A DT R V D L TL L R T I P	Otolemur garnettii
XP_008838446.1 (+)	TQILQ L S D V S D I V VR V L L P V L M	Nannospalax galili
XP_004598397.1 (+)	G M A R A N V D N I V C G R T T I V M NC L VP	Ochotona princeps
XP_026352388.1 (+)	VQ A D A V S H RKR R H I P N	Ursus arctos horribilis
XP_008704652.2 (+)	VQ A D A V S H RKR R H I P N	Ursus maritimus
XP_031535452.1 (+)	A T A R D E A V W T R H I R NP V	Vicugna pacos
XP_034519429.1 (+)	V A D A V S H WKR R H I P N	Ailuropoda melanoleuca
XP_017512446.2 (+)	TAQ A L D V S A G W S H I P V NP	Manis javanica
XP_038202866.1 (+)	SH Q L A D S H I A V R QV L L GP S H K	Arvicola amphibius
XP_041515533.1 (+)	SH Q L A D S H I A V R QVG L L GP S H K	Microtus oregoni
XP_004462362.1 (+)	TGA A SV R DRV H AS V L A V VM R RR I R A P M	Dasypus novemcinctus
XP_005344162.1 (+)	SH Q L A D S H I A V R QV L L GP S H K	Microtus ochrogaster
XP_020844728.1 (+)	S K A D S N G V A F I R A F T N GR S DITAF	Phascolarctos cinereus
XP_024899956.1 (+)	Q VA V D VF R A V A R V H I N A P	Pteropus alecto
XP_027814539.2 (+)	A A A D V A W R S H T FI R D NP	Ovis aries
XP_034355285.1 (+)	IH Q L D S D V I V R R QV L L GP S LRK	Arvicanthis niloticus
XP_036609264.1 (+)	S K A D K D N G V A F I A I F T N GR G DVTDF	Trichosurus vulpecula
XP_021014715.1 (+)	TQ Q L D S D V I V R R QV L L GP S FRK	Mus caroli
XP_027731548.1 (+)	D K A D N G V A F I A F T N GR G DITAF	Vombatus ursinus
XP_008057448.1 (+)	G A A YT R DRL S A T A V K R R I A V IP V	Carlito syrichta
XP_003476372.1 (+)	TK VA S DRV E T TF V VQ M LV E	Cavia porcellus
XP_021503727.1 (+)	TQ Q L V D S D I V I V R Q HV L L GP S	Meriones unguiculatus
XP_028619281.1 (+)	TQ Q L D S D V I V R R QV L L GP S LRK	Grammomys surdaster
XP_003503308.1 (+)	TQ Q L A D S D I A V R R QV L L GP S LRK	Cricetulus griseus
XP_006232550.1 (+)	TQ Q L D S D V I V M R R QV LV L GP S LRK	Rattus norvegicus
XP_036045530.1 (+)	TQ TQ L A D S D I A V R R QV L L GP S LEK	Onychomys torridus
XP_003766246.1 (+)	S K A M D D N SS V A F S V S F TL N GR G S V DIIAF	Sarcophilus harrisii
XP_021052694.1 (+)	TQ Q L D S D I V I V R R QV L L GP S FRK	Mus pahari
XP_005076184.2 (+)	TP Q L A T D S D A V R R QV L N R L GP S L K	Mesocricetus auratus
XP_031231888.1 (+)	TQ Q L D S D V I V R R QV L L GP S FRK	Mastomys coucha
NP_001018041.2 (+)	TQ Q L V D S D V I V R R QV L L GP S FSK	Mus musculus
XP_006972712.1 (+)	TQ TQ L A V D S D I A V R R QV L L GP S LEK	Peromyscus maniculat...
XP_036762030.1 (+)	TAQ A L D V S A W S H I P V NP	Manis pentadactyla
XP_004704163.1 (+)	G A A A GV DRV P H S RV A L R R I N A PT	Echinops telfairi
XP_028723080.2 (+)	TQ TQ L A V D S D I A V R R QV L L GP S	Peromyscus leucopus
XP_040830878.1 (+)	G I A A N V D I V C G R T IV N M A NC L VP	Ochotona curzoniae
XP_028930271.1 (+)	S A D E ED N I T V N K LART S L T F T NV GR L V	Ornithorhynchus anatinus
XP_038600658.1 (+)	S T P S F E ED N I R I A M H K LART S L F T NV GR L T N V	Tachyglossus aculeatus
