# Supplementary material for: Natural Mutations Affect Structure and Function of gC1q Domain of Otolin-1
Source: Int J Mol Sci. 2021 Aug 23;22(16):9085. doi: 10.3390/ijms22169085 (PMC8396674; doi:10.3390/ijms22169085)
Supplement: Supplementary file 1 [file ijms-22-09085-s001.zip › Supplementary File 2.pdf]

Supplementary material

Natural mutations affect structure and function of gC1q domain of otolin-1

Rafał Hołubowicz*, Andrzej Ożyhar, Piotr Dobryszycki*

Department of Biochemistry, Molecular Biology and Biotechnology, Faculty of Chemistry, Wrocław University of Science and Technology, Wybrzeże Wyspiańskiego 27, 50-370 Wrocław, Poland
* - Corresponding Authors
R.H.: phone +48 71 320 63 34, e-mail rafal.holubowicz@pwr.edu.pl
A.O.: phone: +48 71 320 63 33, e-mail andrzej.ozyhar@pwr.edu.pl
P.D.: phone +48 71 320 63 32, e-mail piotr.dobryszycki@pwr.edu.pl
ORCID IDs: R.H: 0000-0002-2555-2069; A.O.: 0000-0002-5874-784X; P.D.: 0000-0001-5522-2557

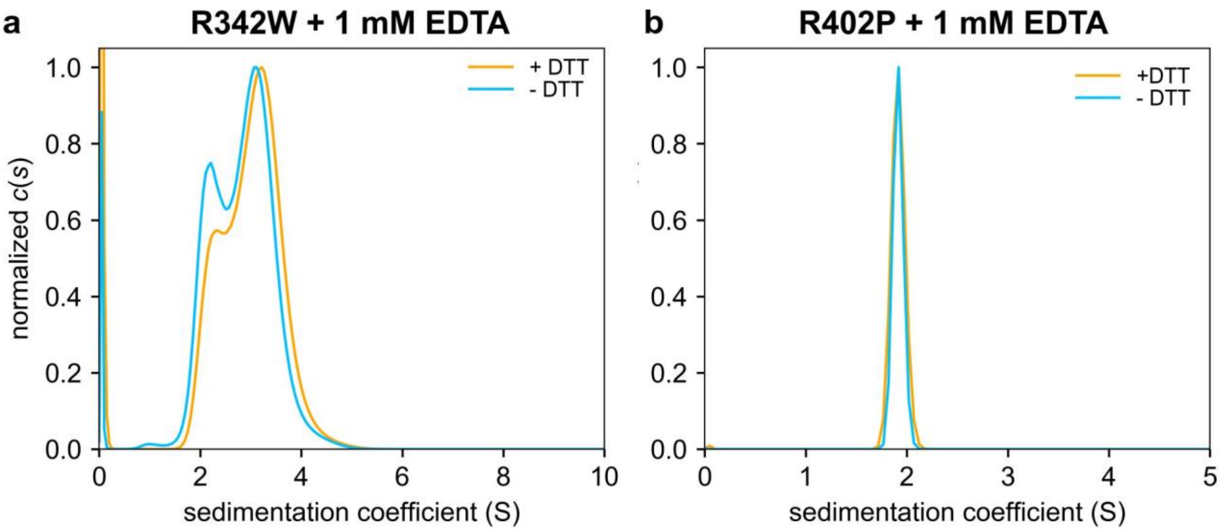

Figure S1. Dithiothreitol (DTT) does not affect the oligomerization of hOtolC1q R342W and R402P. Normalized sedimentation coefficient distributions ($c(s)$) calculated from the sedimentation velocity analytical ultracentrifugation (SV AUC) data collected for (**a**) hOtolC1q R342W and (**b**) hOtolC1q R402P in the absence of Ca^{2+}, with or without 1 mM DTT.

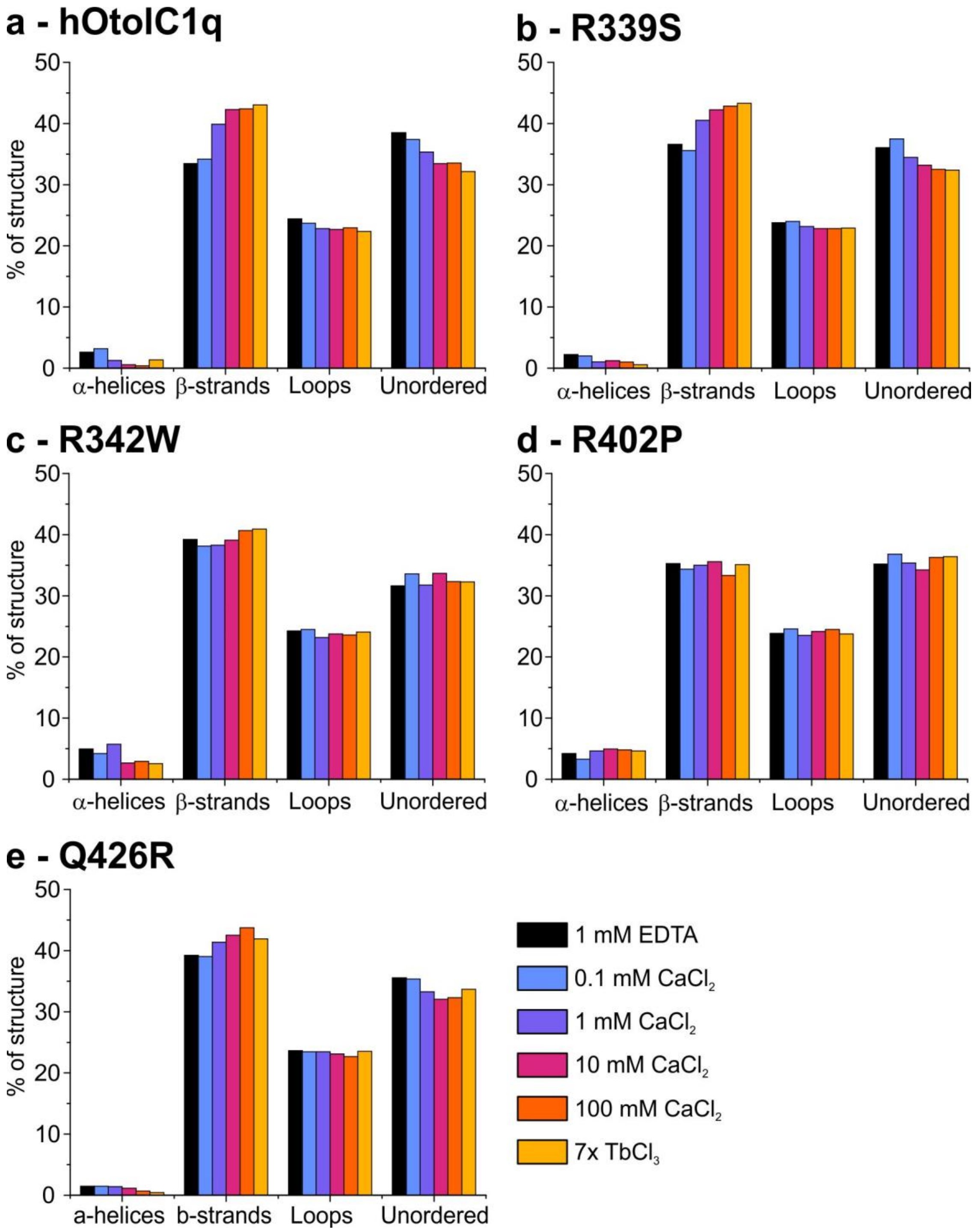

Figure S2. Estimation of the secondary structure content of hOtolC1q and its mutants. The analysis for (**a**) hOtolC1q, (**b**) R339S, (**c**) R342W, (**d**) R402P and (**e**) Q426R was done based on the CD spectra collected in the presence of EDTA, $CaCl_2$ or $TbCl_3$ using CDPro suite applications CDSSTR, CONTINLL, SELCON3 using a reference ser 10 (SMP56 – 43 soluble proteins and 13 membrane proteins).

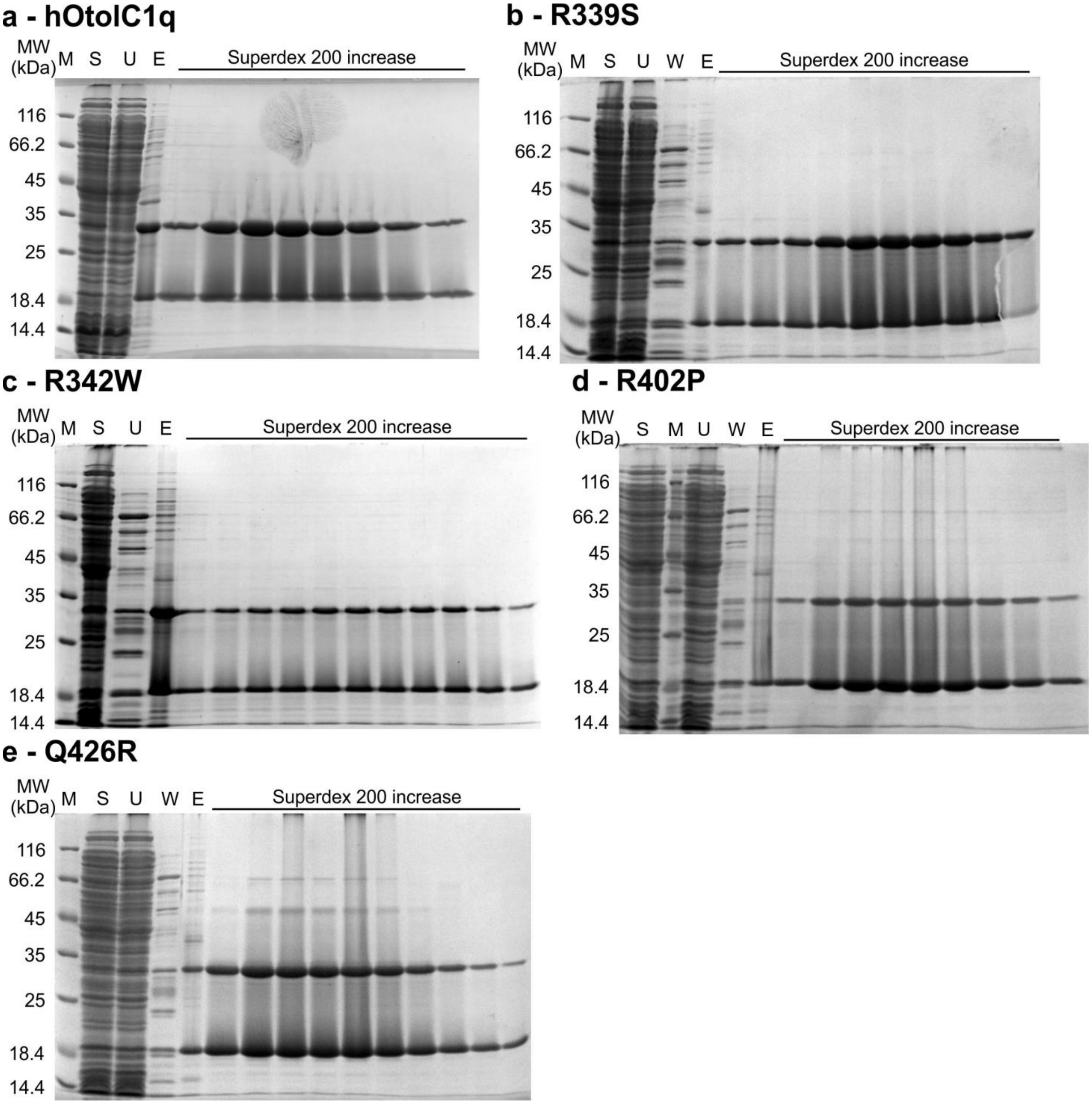

Figure S3. Purification of hOtolC1q and its mutants. The samples of (**a**) hOtolC1q, (**b**) R339S, (**c**) R342W, (**d**) R402P, (**e**) Q426R were collected during the purification and subjected to SDS-PAGE in reducing conditions. M – molecular weight marker, S – supernatant after lysis, U – proteins which did not bind to the TALON Metal Affinity Resin, W – proteins washed away at intermediate imidazole concentration, E – proteins eluted from the metal affinity resin and concentrated before the gel filtration on the Superdex 200 increase column.

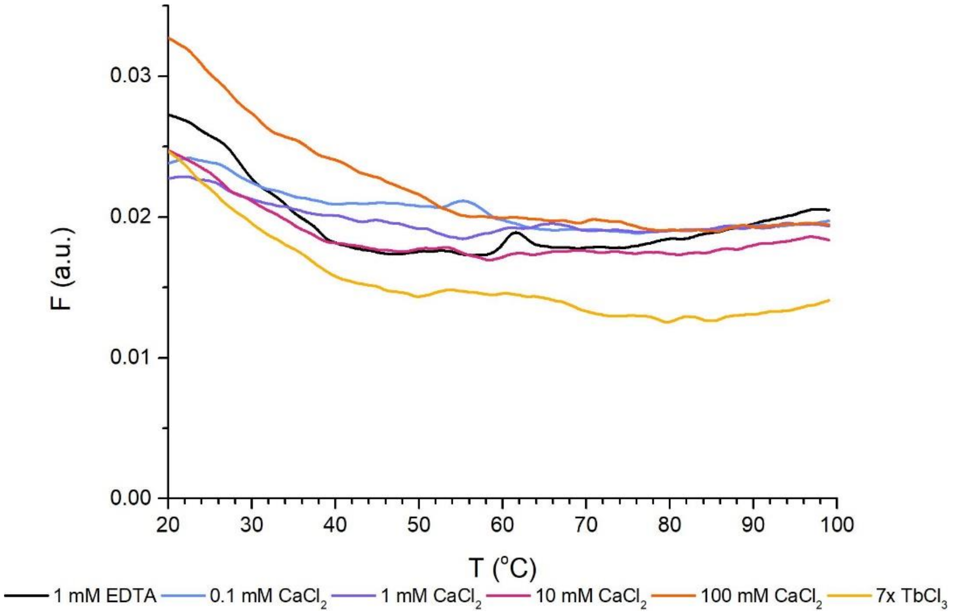

Figure S4. Background fluorescence in the thermal shift assay. Protein-free controls with the additives were subjected to the thermal shift assay on the same 96-well plate as the tested protein samples.

Table S1. Parameters derived from the sedimentation velocity analytical ultracentrifugation. The table contains solvent-corrected sedimentation coefficients ($s_{20,w}$), weight averaged sedimentation coefficients ($\overline{s_{20,w}}$), apparent molecular weights (MW_{app}) and frictional ratios (f/f_0). c – protein concentration, n – stoichiometry of the oligomer. Molecular weight of hOtolC1q and mutants is approximately 17 kDa (estimated using ProtParam tool - https://web.expasy.org/protparam/).

Protein	c [mg/ml]	$s_{20,w}$ [S]			$\overline{s_{20,w}}$ [S]			MW_{app} [kDa] (n)			f/f_0			RMSD		
		1 mM EDTA	10 mM $CaCl_2$	7x $TbCl_3$	1 mM EDTA	10 mM $CaCl_2$	7x $TbCl_3$	1 mM EDTA	10 mM $CaCl_2$	7x $TbCl_3$	1 mM EDTA	10 mM $CaCl_2$	7x $TbCl_3$	1 mM EDTA	10 mM $CaCl_2$	7x $TbCl_3$
hOtolC1q	0.10	3.19	3.46		3.20	3.46		38.5 (2.3)	44.9 (2.6)		1.27	1.30		0.0056	0.0055	
	0.25	3.24	3.46	3.55	3.25	3.47	3.55	40.4 (2.4)	45.5 (2.7)	49.0 (2.9)	1.29	1.31	1.34	0.0059	0.0057	0.0050
	0.50	3.30;<br>5.37	3.47		3.40	3.45		41.6 (2.4);<br>86.5 (5.1)	45.5 (2.7)		1.30	1.31		0.0064	0.0064	
R339S	0.10	2.96	3.48		2.96	3.48		35.1 (2.1)	48.4 (2.8)		1.29	1.36		0.0042	0.0041	
	0.25	3.10	3.48	3.52	3.10	3.48	3.52	37.1 (2.2)	48.7 (2.9)	48.6 (2.9)	1.28	1.36	1.35	0.0044	0.0044	0.0050
	0.50	3.18	3.47		3.28	3.47		37.9 (2.2)	48.2 (2.8)		1.26	1.36		0.0050	0.0052	
R342W	0.10	3.07;<br>4.26	3.60		3.68	3.61		34.4 (2.0);<br>56.2 (3.3)	49.0 (2.9)		1.22	1.32		0.0042	0.0043	
	0.25	2.95;<br>4.25	3.54;<br>5.21	4.23	3.81	3.60	4.30	33.5 (2.0);<br>57.8 (3.4)	47.2 (2.8);<br>84.2 (5.0)	57.2 (3.4)	1.25	1.31	1.25	0.0045	0.0048	0.0049
	0.50	2.86;<br>4.32	3.57;<br>5.30		4.08	3.69		31.0 (1.8);<br>57.7 (3.4)	47.6<br>(2.08);<br>86.1 (5.1)		1.23	1.31		0.0056	0.0058	
R402P	0.10	2.58	2.59		2.58	2.59		32.3 (1.9)	32.6 (1.9)		1.40	1.40		0.0043	0.0042	
	0.25	2.58	2.59	2.66	2.58	2.59	2.66	32.7 (1.9)	32.6 (1.9)	31.2 (1.8)	1.40	1.40	1.32	0.0046	0.0046	0.0049
	0.50	2.60	2.61		2.60	2.61		32.7 (1.9)	32.3 (1.9)		1.40	1.40		0.0055	0.0053	
Q426R	0.10	3.25	3.41		3.25	3.41		43.8 (2.6)	45.1 (2.7)		1.36	1.32		0.0041	0.0041	
	0.25	3.30	3.42	3.52	3.30	3.42	3.52	44.7 (2.6)	46.5 (2.7)	48.6 (2.9)	1.36	1.34	1.34	0.0045	0.0046	0.0051
	0.50	3.33	3.44		3.33	3.44		44.3 (2.6)	46.6 (2.7)		1.33	1.34		0.0054	0.0060	

Table S2. Transition temperature (T_m) values (in °C) determined using the thermal shift assay.

	1 mM EDTA	0.1 mM $CaCl_2$	1 mM $CaCl_2$	10 mM $CaCl_2$	100 mM $CaCl_2$	7x $TbCl_3$
hOtolC1q	44.2	46.1	65.9	87.1	98.8	84.2
R339S	43.7	45.2	65.5	86.9	98.3	82.7
R342W	37.1	35.7	35.5	86.9	95.4	75.2
R402P	46.8	47.0	46.8	46.4	46.1	47.1
Q426R	57.2	58.4	61.6	72.2	89.6	85.5
